# Functional Laterality of the Lower Limbs Accompanying Special Exercises in the Context of Hurdling

**DOI:** 10.3390/ijerph16224355

**Published:** 2019-11-07

**Authors:** Janusz Iskra, Ryszard Marcinów, Bożena Wojciechowska-Maszkowska, Mitsuo Otsuka

**Affiliations:** 1 Faculty of Physical Education and Physiotherapy, Department of Sport University of Technology in Opole, 45-758 Opole, Poland; j.iskra@awf.katowice.pl (J.I.); r.marcinow@po.edu.pl (R.M.); 2 Faculty of Sport and Health Science, Ritsumeikan University, 1-1-1 Nojihigashi, Kusatsu 525–8577, Shiga, Japan; otsuka-a@st.ritsumei.ac.jp

**Keywords:** hurdle run, functional asymmetry, hurdle exercises, teaching hurdles

## Abstract

Background: The purpose of this study was to investigate the lateralization of the lead leg during special exercises and the relationship with athletic performance throughout a hurdling session. Methods: Thirty-eight physical education students participated in the study. A novel three-part “OSI” test (walking over hurdles arranged in a circle, spiral, and straight line) was performed, and various hurdle practices (jogging and running) were selected as research tools. The lead leg selected by the participants was taken into consideration, and the relationship between the chosen lead leg and athletic performance in the five tests was established. Results: The lateralization of the lead leg changed depending on the shape of the running course. The results of further analysis showed (i) no correlation between the use of the right leg as the lead leg in three tests conducted at a marching pace, and (ii) a significant positive correlation between tests performed at the marching and running paces. Conclusion: Hurdlers flexibly change the dominant leading leg depending on the shape of the running course. The results of this research could prove helpful in the training of athletes for hurdling competitions, especially young runners in 400-m hurdles involving straight and corner tracks.

## 1. Introduction

The human body, considered in relation to the sagittal plane, is two-sided. Asymmetry can be discussed in terms of morphological and functional aspects. Functional asymmetry is associated with the dominance of one of the cerebral hemispheres and, as a result, the dominance of one of the upper or lower limbs. Quantitative differences, such as the results of performance and jumping tests, are related to the concept of dynamic asymmetry. The expression of functional asymmetry—the selection and dominant use of one of the limbs—is called lateralization. We assessed the degree of lateralization by means of classic human dynamic measurements—for instance, by comparing dynamic characteristics in regard to the right and left limbs [1,2]. This study applied a mixture of observation methods used in sport sciences, among other fields [3,4].

The dominant role of a selected lower limb is often determined by the specificity of a given discipline. Such phenomena can be observed in activities such as martial arts, football, and selected athletic competitions such as jumps or hurdles [5,6,7]. The analysis of the laterality of the lower limbs in conditions associated with sports competitions also applies to aspects not directly related to sports, such as horse racing [8].

Hurdles are a type of athletic competition in which the cyclical nature of the race is mixed with acyclicality because the athletes need to clear 10 hurdles. The asymmetric nature of clearing hurdles involves the problem of whether the hurdler’s right or left leg should initially lead. The “lead leg” is strictly defined for 100/110-m hurdles (only the left or right leg) but can be alternated in the 400-m hurdle race. The rules regarding hurdles in 100/110-m races include the requirement to clear 10 hurdles. Competitors with advanced technical skills try to clear hurdles in a three-step rhythm and consequently attack every hurdle with the same selected lead leg. The three-step rhythm between hurdles presents a considerable challenge for less-advanced athletes. Because less-advanced athletes try to maintain a rhythm involving four steps, they clear obstacles by alternating between the right and left leg [9,10,11]. Only a few competitors in the history of the 400-m hurdle competition (at the highest, world-class level) have been able to complete the race by attacking the hurdles with only a one-sided lead leg [12]. Most competitors alternate their lead leg during the race, which is often associated with the need to decrease their pace in subsequent stages of the sprint [13,14,15]. Hurdling around a curve in 400-m hurdles seems to be particularly difficult. This challenge is related to the athletes leaning their weight sideways into the center of the curve to counteract the centrifugal force [16]. When acquiring hurdling skills, training to clear hurdles using either leg is important [17,18]. Similar to jumping competitions (advancing using the left or right leg), some people use a specific lead leg for hurdle clearance in hurdles [2], and some practice this movement during classes with special exercises that are performed at a marching or jogging pace. The importance of including such exercises in the teaching process has been confirmed in scientific reports [10].

### 1.1. Objective of the Study

The objective of the present study was to compare the lateralization of the lead leg during special exercises performed at a marching or jogging pace with that of the lead leg during hurdle clearance at standard distances for a specific level of competition at a maximal pace.

### 1.2. Research Questions

This study aimed to answer three research questions:

1. Are there differences between the lateralization of the lead leg selected in three tests that involve clearing hurdles at a marching pace, taking into account different directions of exercise practice?
2. Are there differences in terms of the lead-leg lateralization of the subjects among five tests that involve clearing hurdles at marching, jogging, and maximal paces?
3. Are there correlations between the functional asymmetries of the selection of the right or left leg as the lead leg during hurdle clearance at marching, jogging, and maximal paces?

### 1.3. Hypotheses

The following hypotheses were proposed:

1. There are no differences in the lateralization of the lead leg selected by participants in three tests involving marches over hurdles.
2. There are no differences in the lead leg selected by participants in all forms of hurdle exercises.

## 2. Materials and Methods

### 2.1. Participants

The study involved a group of students with a specialty in motor skill training at the Faculty of Physical Education and Physiotherapy at the Opole University of Technology (Table 1). The group comprised 12 women (age: 23.23 ± 2.07 years; body weight: 57.47 ± 3.57 kg; body height: 1.66 ± 3.24 m) and 26 men (age: 24.24 ± 2.11 years; body weight: 75.81 ± 5.11 kg; body height: 181.79 ± 4.45 m). None of the participants were professional hurdlers; all were students of physical education. We did not divide the group into males and females, as many authors have shown that both males and females encounter the same problem of choosing a so-called hurdle stride pattern “rhythm” [16,19,20]. The tests were carried out in an athletic sports hall on a tartan track. The first test started in the late morning (i.e., from 11:00 a.m.), and subsequent tests were performed on the same day at half-hour intervals. The subjects voluntarily agreed to participate in the research and were informed of the purpose of the study. In this group, there were no professional athletes (hurdlers). There were no reasons to exclude any students from participating in this study. The whole teaching session was typical of physical education for students.

### 2.2. Study Procedure

In our study, we used an observation method that included a mixed method of analysis [3]. After observing many hurdling exercises, we counted left and right movements and converted the values into percentages, which were subjected to statistical analysis.

The laterality of the lead leg was assessed using the five trials (specialist tests) described below.

#### 2.2.1. “OSI” Test: A Novel Test Developed in this Study

**Figure 1:**
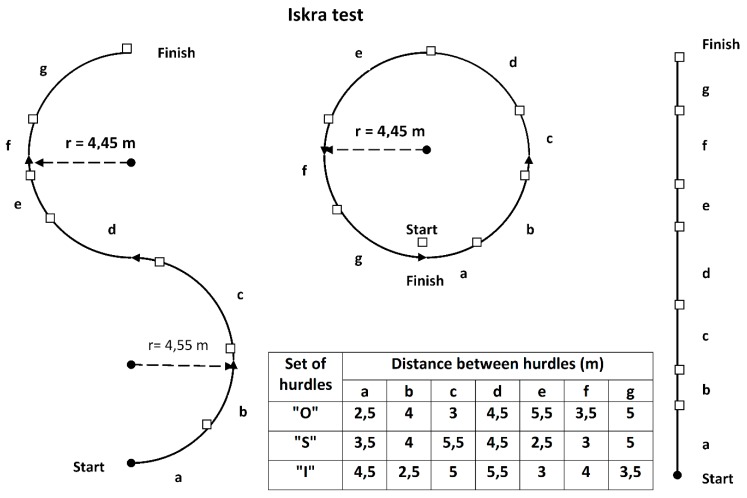
Hurdle arrangement (a square denotes a hurdle) and the intervals between hurdles for tests performed in a circle, on a curve, and on a straight track.

The subjects had to clear hurdles (76 cm for women, 91 cm for men) arranged at uneven intervals and along various trajectories: in a straight line, along the circumference of a circle, and along S-shaped curves. The distances between hurdles (from 2.5 to 5.5 m, every 0.5 m) were determined randomly for each test (Figure 1). The subjects stood on the starting line with their legs in a step position. The subjects did not receive any advice regarding the starting position or technique for clearing the hurdles. While the subject performed the tests, the selection of the lead leg (left or right) was recorded for each hurdle clearance.

**Test no. 1**: March along a circle, "O". The subject started the test at the start/finish line. At the signal of the coach, the subject began to march around the circumference of the circle in a counterclockwise direction. An additional final hurdle was placed at the start after the subject began the lap. The radius of the circle and the distances between hurdles are specified in Figure 1.

**Test no. 2**: March along a curved track, "S". The subject started the test at the beginning of the curve, which curved in a counterclockwise direction. In the second part of the curve, the direction of the march shifted to clockwise. The radius of the curve and distances between hurdles are shown in Figure 1.

**Test no. 3**: March along a straight track, "I". The distances between the hurdles are displayed in Figure 1. The distance in a maximal hurdle run is standard for students of physical education.

There was a main difference between hurdle clearance on a straight track and that in a run around the track. When teaching the 400-m hurdle run, we applied exercises (mainly at a marching pace) on various movement tracks (circle slalom). For this distance, we used an “anticlockwise” direction for the run with eight variants (eight tracks with different curvatures). 

#### 2.2.2. Two Tests Involving Hurdle Clearance at Jogging and Sprinting Paces

**Test no. 4**: This test involved a light jog covering a distance of 50 m and the clearing of hurdles with a height of 76 or 91 cm (for women and men, respectively). The subject started the test at the start/finish line (legs in a step position). When the coach provided a signal, the subject began clearing the hurdles, attacking them with the leg of their choice. While the subject performed the trial, the selection of the lead leg (left or right) was recorded for each hurdle that was cleared. The subject did not receive any advice regarding the starting position or technique for clearing the hurdles. The distances between the hurdles were set randomly (from 4 to 10 m). The velocity of this exercise (test) was determined as “medium run/jog”. The time of the test was 14.98 ± 1.48 s (ranging from 12.54 to 17.98 s) (Table 1).

**Test no. 5**: This test involved sprinting (at maximal velocity) for 60 m and clearing hurdles with a height of 76 or 91 cm (for women and men, respectively). The subject started the test at the start/finish line (legs in a step position). At the signal of the coach, the subjects began to run, clearing the hurdles with the leg of their choice. The subject did not receive any advice regarding the starting position or the best manner for clearing the hurdles. While the subject performed the test, the lead leg (left or right) was recorded for each hurdle that was cleared. The distances between the hurdles were set to 7.50 m for women and 8.20 m for men (in both cases, these distances are equal to approximately 4.5 times the average body height of the subjects participating in the study) following the study by Iskra and Mynarski [10]. The approach (i.e., the distance from the start to the first hurdle) was 12 or 13 m. This run was the final run conducted for students after the hurdle course. In the protocol in this test, the time of the run was 10.85 ± 0.94 s (from 9.20 to 12.98 s).

### 2.3. Statistical Analysis

The results were analyzed using Statistica 13.1 software (TIBCO Sofware Inc., Tulsa, OK, USA). The significance level was set to *p* ≤ 0.05. The normality of the distribution was assessed by the Shapiro–Wilk test. Student’s *t*-test was used to analyze the differences in the selection of the left or right leg in the trials involving hurdle clearance along a curve and along a straight line, whereas the differences in the results based on the arrangement of hurdles (circle, curve, and straight line) were assessed by analysis of variance (ANOVA). The analysis of the dependencies between the leg selection patterns in the trials performed at a marching pace and those performed at jogging and running paces were performed using Spearman’s Rank correlation coefficient. The Cohen effect sizes in all statistical tests were determined using G*Power 3.1 (Heinrich-Heine-Universität Düsseldorf, Düsseldorf, Germany). Tests for correlation and regression analyses [21]: d = 0–0.1 was considered as no effect, 0.2–0.4 as small effect, 0.5–0.7 as intermediate effect and ≥0.8 as large effect. Similarly, partial η^2^ effect size: 0.01–0.06 small, 0.06–0.14 medium and ≥0.14 large effect.

## 3. Results

In this study, we used an observation method that included a mixed method of analysis [3]. After observing many hurdle exercises, we counted left and right movements and converted the values into percentages, which were then subjected to statistical analysis.

Table 2 shows the mean results for the lead leg selected by subjects in tests in which they cleared hurdles at a marching pace. In all tests, the subjects had a greater tendency to use the right leg to clear the hurdles, with a large difference between the maximum and minimum results. The analysis of the results demonstrates that there were even cases in which subjects cleared all the hurdles with the right leg. Such cases were not recorded for the left leg.

Table 2 contains a summary of the mean results for lead leg selection in the tests in which the subjects cleared the hurdles at jogging and running paces. Similar to the tests in which the subjects cleared the hurdles at a marching pace, a preference for the right leg was observed in the tests performed at jogging and running paces. The results of test nos. 4 and 5 also demonstrate cases in which the subjects cleared all hurdles with the right leg. Cases in which the subject cleared all hurdles with the left leg were only observed in test no. 5 (i.e., the test that was closest to the conditions of an actual competition).

Table 3 presents a comparative analysis of the mean results of the tests performed at a marching pace. The analysis shows statistically significant differences between the selection of the left and right leg as the lead leg in trials involving hurdle clearance around a curve and along a straight line. In these tests, the subjects more frequently used their right leg as the lead leg. In the test in which participants performed the hurdling test in a circle at a marching pace, the difference was not statistically significant. The mean totals in all three trials performed by subjects at a marching pace revealed statistically significant differences in the choice of lead leg. The majority of the subjects cleared the hurdles with the right leg more frequently than they used the left leg. Such differences were not observed in the hurdling test performed in a circle at a marching pace.

A comparative analysis of the mean results of the test performed at the jogging and running paces (Table 3) demonstrated that the differences in the selection of the left or right leg were statistically significant. Similar to the tests performed at a marching pace, in the trials at the jogging and running paces, the subjects more frequently selected the right leg as the lead leg.

The selection of the right leg was preferred in four of the five cases. However, an additional question remained: In attempts to clear hurdles at a marching pace, does the arrangement of the hurdles (circle, curve, or straight line) affect the mean frequency at which the right leg is selected? The ANOVA did not reveal such differences (Table 4). The mean results were rescored on the basis of the attempts to clear hurdles with the right leg (i.e., for various arrangements of the hurdles), and these results proved to be similar and were not significantly different. This collection of data proves that the trajectory of the track affected the selection of the limb used for hurdle clearance.

Table 5 contains the statistical analysis results for the relation between the lead leg selection in the tests performed at a marching pace (test nos. 1, 2, and 3) and the lead leg selection in the tests performed at jogging and sprinting paces (test nos. 4 and 5). Interestingly, the results did not show a correlation based on the hurdle arrangement in the test performed at a marching pace. The results of all trials that were performed at a marching pace were only correlated with the final result of other tests performed at a marching pace. The results of the remaining tests were not significantly correlated with each other. The results of the lead leg selection in the tests performed on curved tracks (circle, spiral) did not correlate with the results of the hurdle clearance tests performed at jogging and sprinting paces, whereas the result of the lead leg selection in the straight line test was significantly correlated with the result of the lead leg selection in the tests performed at a jogging pace (0.64 for *p* ≤ 0.05). The results of the selection of the lead leg in the “OSI” test correlated with the lead leg selection results in the test performed at a jogging pace (0.58 for *p* ≤ 0.05) and did not affect the selection of the lead leg in the test performed at a sprinting pace (0.47 for *p* ≤ 0.05). A significant level of dependence was also observed between the selection of the lead leg during the hurdle clearance in the tests performed at jogging and sprinting paces (0.58 for *p* ≤ 0.05). Thus, significant dependencies can be established for exercises performed at similar speeds (march–jog and jog–run). Significant speed differences (march–run) resulted in the subjects varying the manner in which they cleared the hurdles.

## 4. Discussion

The problem of lateralization in physical education and sports is important in various activities. Analyses of the functional dominance of the limbs, especially the lower limbs, have shown four types of laterality: lateralization combined with right leg dominance (type I) and left limb dominance (type II), right-footedness, and left-footedness [22]. The dominance of a particular leg can be assessed in various ways, from questionnaires to specific physical tests [23]. The term “specific” in this study also applies to hurdles.

In this study, we did not account for the potential influence of motor preparation on the choice of lead leg and, more broadly, the choice of stride pattern. This problem has been addressed in many previous studies [24]. Speed, endurance, and strength are known to be connected to the strategy used in the 400-m hurdle run, but this is a separate problem for another study.

Hurdle competitions include two athletic events—the 100/110 m and the 400 m—that differ in terms of the specialized effort required (focused on speed and speed-endurance, respectively). Analysis of the results of all tests demonstrated that the subjects more frequently used the right leg to clear the hurdles in the test performed at a running pace ($\bar{x}$ = 4.53 m/s), whereas the selection of the right leg as the lead leg was least common in the hurdle test performed in a circle at a marching pace ($\bar{x}$ = 3.89 m/s). Similarly, the preference of the right leg as the lead leg is common in the 100/110-m hurdle race, with over 60% of competitors participating in the most prestigious athletic events preferring to use their right leg as their lead leg [16].

The analysis of the results showed that the subjects participating in the test cleared hurdles using the right leg as the lead leg more frequently during trials performed on straight track sections. However, this finding differs from the results of the hurdle test performed in a circle. The direction of the running movement followed the direction of the race in athletic competition conditions (i.e., counterclockwise). This may indicate that some of the subjects who normally used their right leg as their lead leg in the other tests on a circular track decided to select the left leg for the test involving a counterclockwise run. This observation supports the results reported in earlier studies [16,19], which demonstrated that subjects find it easier to clear hurdles with the inner left leg (as the body adapts to counteract the centrifugal force) during hurdle clearance on a curve. In fact, Starosta and Kędziora [25] found that the 400-m hurdle race specifically causes the lead leg to change, regardless of the leg that is normally preferred.

In test no. 5 (hurdle clearance at the maximal running pace), the distance between hurdles was set to approximately 4.5 times the mean body height, which is the distance used for beginners. At such distances, novice athletes should (in conditions similar to competition conditions) be able to clear hurdles in a three-step rhythm without attacking each hurdle with the same leg [11,26,27]. However, among the 38 subjects in the study, only 21 cleared all hurdles with the same leg (i.e., taking an odd number of steps between hurdles), and the other subjects changed their lead leg for the task of hurdle clearance. These results could be attributable to issues that extend beyond lateralization. As has been repeatedly stated, the selection of the lead leg in hurdles can also be determined by the level of motor skills (including speed, glycolytic endurance, and leg strength) [28].

The shorter-distance race is performed on only a straight track, as opposed to longer tracks that include straight sections, curves, and arched parts; this difference leads to variation in technical skills between hurdlers [19,29]. Analysis of the correlation for the marching-pace tests confirms this conclusion. The results of the marching-pace test performed on a straight track correlated with the results of the test involving jogging and running along a straight track, but such a correlation could not be established between the lead leg selection with hurdles arranged on a straight track and that with hurdles arranged around curves (with different hurdle arrangements and moving either clockwise or counterclockwise), as shown in Table 5. The alternating selection of the lead leg due to the arrangement of hurdles has often been attributed to fatigue related to anaerobic exercise [28,30]. The results of this study suggest an association with the direction/track followed during a hurdle test. Hurdling exercises performed at a marching pace demonstrate that the technical exercise of hurdlers plays a significant role [19,20,29]. Research has shown that this approach to the development of technical skills is closely related to hurdle performance (both in the form of jogging and running). Therefore, performing hurdle exercises at a marching pace can have a considerable impact on the progression of an athlete’s hurdling technique. 

In this study, we used an observation method that included a mixed method of analysis [3]. After observing many hurdle exercises, we counted left and right movements and converted the values into percentages, which were then subjected to statistical analysis.

High clearance at high (jogging) and maximal velocity (hurdle sprinting and running) was more difficult than special hurdle exercises at a marching pace. This difficulty is likely the reason that students chose the right leg to “jump” over hurdles. They considered (contrary to standard teaching for all hurdlers–from beginners to professional) this decision to be not only effective but also safe.

In previous studies, we did not find relationships between right (dominant) handedness and lead leg preference in a hurdle run [31].

The research was limited by a group of physical education students who did not specialize in professional hurdling, so our results cannot be generalized to professional athletes.

## 5. Conclusions

The hypotheses in this study were confirmed only partially.

1. From all tests involving hurdle clearance (marching, jogging, and running), the study demonstrates the dominance of the right leg. Therefore, the left leg can be considered the dominant take-off leg.
2. The lack of a correlation between the leg selection at the marching pace and that at the running pace demonstrates that the specific approach followed during hurdle clearance depends on the profile of the track.
3. The results of tests involving hurdle clearance at various speeds (marching, jogging, and running) demonstrate a correlation only between tests conducted at similar speeds (march–jog and jog–run). Such similarities between tests were not observed when hurdling clearance was performed at considerably different speeds (march–run).
4. The results of this research could prove helpful in the teaching hurdling running to young athletes, especially over longer distances, taking into account races following a curve (i.e., after a distance of 150–300 m in hurdling races).

## Figures and Tables

**Table 1:** The arrangement of hurdles in the test performed at jogging and running paces.

Test		Distance Between Hurdles (m)						
		a	b	c	d	e	f	g
Test 4		4.0	5.0	6.0	7.0	8.0	9.0	10.0
Test 5	F	12 (A)	7.5					
	M	13 (A)	8.2					

(A): approach.

**Table 2:** Lead-leg functional asymmetry in tests involving hurdle clearance at a marching pace (data in %) and the results of tests performed in a jog and a run.

Test No.	Type Of Test	Lead Leg	Mean ($\bar{x}$)	Stand. Dev. (SD)	Min.	Max.	Skewness	Kurtosis
1	March in a circle “O”	L	3.11	1.20	0	6	0.28	1.22
		R	3.89		1	7	−0.28	
2	March around a bend “S”	L	2.53	1.75	0	6	0.11	−0.44
		R	4.47		1	7	−0.11	
3	March in a straight “I”	L	2.63	1.60	0	6	−0.11	−0.24
		R	4.37		1	7	0.11	
Total OSI		L	8.26	3.64	0	18	0.47	1.47
		R	12.74		3	21	−0.47	
4	Hurdle jog	L	2.71	1.59	0	6	−0.17	−0.66
		R	4.29		1	7	0.17	
5	Hurdle run	L	2.47	2.50	0	7	0.81	−0.49
		R	4.53		0	7	−0.81	

**Table 3:** Student’s *t*-test results for the selection of the right and left lead legs during tests at a marching pace and at jogging and running paces (data in %).

Test No.	Type Of Test	Left Leg, L	Right Leg, R	Stand. Dev. (SD)	t	p	d
1	March in a circle “O”	44.36	55.64	17.19	−2.02	0.051	0.57
2	March around a bend “S”	36.09	63.91	25.02	−3.42	0.002 *	2.97
3	March in a straight line “I”	37.59	62.41	22.87	−3.34	0.002 *	7.67
	Total OSI	39.35	60.65	17.35	−3.78	0.000 *	6.14
4	Hurdle jog	38.75	61.28	22.75	−3.06	0.004 *	0.99
5	Hurdle run	35.34	64.66	35.73	−2.52	0.016 *	0.96

* *p* ≤ 0.05; d: Cohen effect size.

**Table 4:** Differences in the results of the tests involving hurdle clearance using the right leg for various hurdle arrangements (data in %).

Hurdle Arrangement	Layout “O”	Layout “S”	Layout “I”	ANOVA		
				F	*p*	*partial* *η* ^2^
Lead leg R ($\bar{x}$/SD)	55.64 (±17.18)	63.91 (±25.02)	62.41 (±22.87)	1.53	0.22 (NS)	0.05

NS: lack of statistical significance; partial η^2^-effect size

**Table 5:** Spearman’s Rank correlation of the trial results for hurdle clearance with the right leg at marching, jogging, and running paces.

Test	March “O”	March “S”	March “I”	Total “OSI”	Jog	Run
March “O”						
March “S”	0.18					
March “I”	0.26	0.46 *				
Total “OSI”	0.50 *	0.80 *	0.81 *			
Jog	0.11	0.45	0.64 *	0.58 *		
Run	0.30	0.33 *	0.44 *	0.47 *	0.58 *	

* *p* ≤ 0.05.

## References

[1] Wolański N. (2005). Human biological development. Rozwój Biologiczny Człowieka.

[2] Hewit J., Cronin J., Hume P. (2012). Multidirectional leg asymmetry assessment in sport. Strength Cond. J..

[3] Anguera M.T., Camerino O., Castaner M., Sanchea-Algarra P., Onwuegbuzie A.J. (2017). The specificity of observational studies in physical activity and sports sciences: Moving forward in mixed methods research and proposals for achieving quantitative and qualitative symmetry. Front. Psychol..

[4] Pic M. (2018). Performance and Home advantage in handball. J. Hum. Kinet..

[5] Stokłosa H. (1994). Functional body asymmetry in experienced weight lifters and wrestlers. Biol. Sport.

[6] Eikenberry A., McAuliffe J., Welsh T.N., Zerpa C., McPherson M., Newhouse I. (2007). Starting with the “right” foot minimizes sprint start time. Acta Psychol..

[7] Hoffman J.R., Ratamess N.A., Klatt M., Faigenbaum A.D., Kang J. (2007). Do bilateral power deficits influence direction-specific movement patterns?. Res. Sports Med..

[8] Williams D.E., Norris B.J. (2007). Laterality in stride pattern preferences in race horses. Anim. Behav..

[9] Hay L., Schoebel P. (1990). Spatio-temporal invariants in hurdle racing patterns. Hum. Mov. Sci..

[10] Iskra J., Mynarski W. (2000). The influence somatic and motor fitness on hurdle results by untrained boys aged 11–15. J. Hum. Kinet..

[11] Otsuka M., Ito M., Ito A. (2010). Analysis of hurdle running at various interhurdle distances in an elementary school PE class. Int. J. Sport Health Sci..

[12] Quercetani R.L. (2000). Athletics: A History of Modern Track and Field Athletics (1860–2000): Men and Women.

[13] Ditroilo M., Marini M. (2001). Analysis of the race distribution for male 400 m hurdlers competing at the 2000 Sydney Olympic Games. New Stud. Athl..

[14] Quinn M.D. (2010). External effects in the 400-m hurdles race. J. Appl. Biomech..

[15] Otsuka M., Isaka T. (2019). Intra-athlete and inter-group comparisons: Running pace and step characteristics of elite athletes in the 400-m hurdles. PLoS ONE.

[16] Iskra J., Cóh M. (2011). Biomechanical Studies on Running the 400 M Hurdles. Hum. Mov..

[17] Thompson P. (2009). The Official IAAF Guide to Teaching Athletics.

[18] Gasilewski J., Iskra J. (2011). Efekty nauczania biegu przez płotki w aspekcie treningu motorycznego i technicznego. Antropomotoryka.

[19] McFarlane B. (2000). The Science of Hurdling and Speed.

[20] Arnold M. (1992). Hurdling.

[21] Faul F., Erdfelder E., Buchner A., Lang A.G. (2009). Statistical power analyses using G*Power 3.1: Tests for correlation and regression analyses. Behav. Res. Methods.

[22] Gabbard C., Hart S. (1996). Foot laterality in children, adolescents and adults. Laterality.

[23] Chapman J., Chapman L., Allen J. (1987). The measurement of foot preference. Neuropsychologia.

[24] Ozaki Y., Ueda T., Fukuda T., Inai T., Kido E., Narisako D. (2019). Regulation of stride length Turing the approach run in the 400-m hurdles. J. Hum. Kinet..

[25] Starosta W., Kędziora R., Kowalski P., Migasiewicz J. (1995). Methods of clearing hurdles by the best male and female hurdlers from the perspective of laterality. Problemy Badawcze w Lekkoatletyce.

[26] Iskra J., Bacik B., Król H., Raczek J., Waśkiewicz Z., Juras G. (2000). The effect of specific exercises on changes in hurdle technique. Current Research in Motor Control.

[27] Hagen R., Trebels A.H. (1998). Hȕrdenlauf—Ein individuelles Rhythmusproblem. Sportpȁdagogik.

[28] Zauhal H., Jabbour G., Jacob C., Duvigneau D., Botcazou M., Ben Abderrahaman A., Prioux J., Moussa E. (2010). Anaerobic and aerobic energy system contribution to 400-m flat and 400-m hurdles track running. J. Strength Cond. Res..

[29] Iskra J. (2014). 400 m hurdlerstraining. Trening Płotkarzy na 400 m.

[30] Ward-Smith A.J. (1997). A mathematical analysis of the bioenergetics of hurdling. J. Sports Sci..

[31] Hyjek J. (2012). Czynniki Warunkujące Rytm w Biegu Przez Plotki Osób o Różnym Poziomie Zaawansowania Sportowego.

